# The status of ethics in Swedish health care management: a qualitative study

**DOI:** 10.1186/s12913-018-3436-8

**Published:** 2018-08-06

**Authors:** Anna T. Höglund, Erica Falkenström

**Affiliations:** 1 Department of Public Health and Caring Sciences, Centre, for Research Ethics and Bioethics, Box 564, SE-751 22 Uppsala, Sweden; 2 SCORE, Stockholm University, SE-106 91 Stockholm, Sweden

**Keywords:** Ethical competence, Health care management, Health care organization, Marketization, Sweden

## Abstract

**Background:**

By tradition, the Swedish health care system is based on a representative and parliamentary form of government. Recently, new management forms, inspired by market principles, have developed. The steering system is both national and regional, in that self-governing county councils are responsible for the financing and provision of health care in different regions. National and local documents regulating Swedish health care mention several ethical values, such as equity in health for the whole population and respect for autonomy and human dignity. It is therefore of interest to investigate the status of such ethical statements in Swedish health care management.

**Method:**

The aim of the present study was to investigate perceptions of the status of ethics in the daily work of politicians, chief civil servants and Chief Executive Officers (CEOs) from care-giver organizations in the county council of Stockholm. A qualitative method was used, based on inductive content analysis of individual interviews with 13 health care managers.

**Results:**

The content analysis resulted in four categories: *Low status of ethics; Cost-effectiveness over ethics; Separation of ethics from management*; and *Lack of opportunities for ethical competence building*. The informants described how they prioritized economic concerns over ethics and separated ethics from their daily work. They also expressed that they experienced that this development had been enforced by the marketization of the health care system. Further, they described how they lacked opportunities for ethical discussions, which could have helped develop their ethical competence.

**Conclusions:**

In order to improve the status of ethics in health care management, ethical considerations and analyses must be integrated in the regular work tasks of politicians, chief civil servants and CEOs; such as decision-making, budgeting and reform work. Further, opportunities for ethical dialogues on a regular basis should be organized, in order to improve ethical competence on the management level. New steering forms, less focused upon market principles, might also be needed, in order to improve the status of ethics in the health care management organization.

## Background

The Swedish health care system is based on a representative and parliamentary form of government. According to the Swedish Health Care Act [1], health care should be democratically governed and solidary financed (i.e., tax based) and care should be given out of need, not, e.g., position or economic status. This is a national legislation, but apart from that there is also self-governance on the local level in Swedish health care, where self-governing regional governments, so called county councils, are responsible for the financing and provision of health care in the region.

Since the beginning of the 21^st^ century, new forms of health care management and administration have been implemented in Sweden. These management forms are to a large extent inspired by the so called New Public Management (NPM). The concept of NPM was coined by Hood [2]. The basic idea is to take governing principles from the private sector and apply them on the public sphere. According to the advocates of NPM, the goal is increased efficiency in the public sector. At the center of NPM is the demand for economic profit, which requires constant measurements and evaluations. Key measures are promoting choice and competition in the health care system.

One example of management inspired by such market principles was the reform regarding Swedish primary care that was introduced in 2010. The reform was based on three principles, namely a “freedom-of-choice-principle” which should make it possible for citizens to choose freely between primary care centers; an “any-qualified-provider-principle” which opened up for private (as well as public) providers to compete for public reimbursements; and a “money-follows-the-patient-principle” which indicated that patients’ choices should impact the allocation of resources. According to responsible politicians this was to be seen as a customer driven model [3].

Alongside the marketization, elected politicians in the county councils are still supposed to take into account the interests of the whole community. The county council politicians are supported by non-elected administrators, who are also responsible for implementing political decisions. This means that the health care management organization on the county council level consists of three key groups of, by tradition, influential actors; namely politicians, chief civil servants and Chief Executive Officers (CEOs) from care giver organizations.

Marketization of health care is built upon the logic of economy, where results and profit are central concepts. This has been transferred to the public sphere; a sphere that is traditionally characterized by a different logic, with values such as democracy and equal rights at its center. Arguably, there is an inbuilt conflict between national equality on the one hand and local self-government on the other in Swedish health policy. Both principles – equality and local self-government - are based in Swedish law, but they can be difficult to harmonize.

The marketization of public health care in Sweden has been much criticized. For example, it has been argued that the logic behind it is suitable for the production of goods and properties, but not value-driven services such as health care [4]. Further, market inspired forms of management has been criticized for increasing bureaucracy in the public sphere - in sharp contrast to its intentions [5]. In response to such criticisms, the current trend is to reduce the administrative demands and thereby the burden of control systems, and to develop more trust-based forms of health care administration and management. This has for example been put forward in the government’s proposed budget for 2015 [6].

The main paragraph in the Swedish Health Care Act states that the goal of Swedish health care is good health and equal care for the whole population. It further says that “care should be provided with respect for all persons’ equal value and the individual person’s dignity” [1].

Also the Patient Security Act [7] and the Patient Act [8] include statements of ethics. The Patient Security Act states that the care provider should work systematically to ensure patient safety and avoid injuries related to care giving. The goal of the Patient Act is to promote patients’ rights in Swedish health care. It prescribes that the care giver must respect the patient’s integrity, autonomy and right to participation in decision-making. No care must be performed without the patient’s consent.

The Swedish parliament has also established guidelines for priority-setting [9], prescribing that priority-setting on all levels in health care should be done according to three ethical principles, namely *the principle of human dignity, the principle of need and solidarity* and, finally, *the principle of cost-effectiveness*. According to the principle of *human dignity* all human beings are equal in dignity, regardless of characteristics and functions in society. Hence, priorities must never be made only on basis of factors such as chronological age, gender or ethnicity. The principle of *need and solidarity* prescribes that resources should be awarded to the patient most in need of them, but also that health care providers should pay special attention to individuals from vulnerable groups, as an act of solidarity. The principle of cost-effectiveness, finally, implies that a reasonable relation between cost and effect should be aimed at in health care providing. This principle is, however, subordinate to the other two.

The National Board of Health and Welfare has published *National Indicators for Good Health Care*, intended to structure the yearly follow-up of health care providing in Sweden [10]. The goal of the indicators, according to the Board, is to increase the quality of care in Sweden. A definition of “good care” is provided, namely that care should be evidence-based, safe and secure, patient-centered, efficient, equal and provided in due time. Such an understanding of good care can be understood as what Goldberg calls “a catalog approach” [11]. The meaning of such a catalog approach is that you identify “specific attributes of care that should be examined in determining its quality” ([11], p., 251). Although such an approach does not offer a definition of “good care” per se, it still provides some guidance for policy-makers and practitioners.

In line with the principles for priority-setting, the indicators further state that need should qualify high priority, and not factors such as age, gender, ethnicity or socio-economic status. However, it also says that there should be a division of responsibility concerning the three principles for priority-setting, in that healthcare personnel are responsible for fulfilling the principle of *human dignity*, while the providers of care (i.e. hospitals or primary care centers) are responsible for the principle of *need and solidarity.* The management level, finally, is responsible for the realization of the principle of *cost-effectiveness* [10].

Apart from these national policy documents, also some regional documents exist. In the county council of Stockholm *a regional ethical code* was established 2002, prescribing fundamental values for the health care provision within the county [12]. The code states that each patient should be treated with *respect* and *dignity*. Further, that patients should be cared for and be shown *consideration* and *concern*. Care should be provided based on *needs*, and the code also says that *justice* is not only about treating people equally and giving care according to needs, but also to use financial resources in an efficient way. Finally, the code also establishes how the presented fundamental values are connected to the management level. It states that the county council should establish possibilities for health care professionals to work according to the ethical code, and that the fundamental values that the code prescribes should be inscribed into agreements and contracts concerning health care providing.

The budget of the county council of Stockholm also mentions ethical values [13]. In the introduction, it says that health care in the county should be accessible for all citizens and that care providing should be safe and efficient. The value of patient-centered care is stated, as well as that care should be provided according to needs, which is in line with the guidelines for priority-setting on the national level. However, as this is a budget, the value of *cost-effectiveness* is much emphasized and priority-settings and reviews of costs are stressed.

In sum, ethical statements seem to be of utmost importance in Swedish health care policy documents, both national and regional. It is therefore of interest to investigate *the status* of such ethical statements in Swedish health care management. Are they merely beautiful words, or are they normative assertions, governing managers’ hard decisions?

### Theoretical framework

Ethics deals with questions such as “What should we do?” and “How should we lead our lives?” Notions of ‘good’ and ‘right’ are at the core of the reasoning. Ethical dilemmas can arise from conflicting values, norms and interests, where there may be good reasons for more than one course of action. A choice has to be made, and the loss of at least one value or interest is unavoidable in severe ethical dilemmas. Hence, ethics is not only about making the right decision in a given dilemma, but also about justifying the decisions and choices made. According to well-established theories, actions are guided as right or wrong according to its consequences, for example in *utilitarian theories*, or according to its’ conforming to ethical rules or duties, so called *deontological arguing* [14].

Deontological and utilitarian theories focus on how to act in ethically difficult situations. Another line of arguing holds that it is *the character* of the moral agent that matters. Such arguing is called *virtue ethics* and a basic assumption in such models is that a good person performs good actions. Virtues, i.e., desirable character traits, such as empathy, courage and patience, are learned through experience, practice, role-models and good examples [15, 16]. Further, self-reflection, dialogue and critical thinking are important traits within the tradition of virtue ethics.

Since the end of the 1900s the so called *ethics of care* has also become influential. Here the experience of giving and receiving care is regarded as morally valuable. The concept of care is regarded both as a practice and as a moral value. Focus is on attending to and meeting the needs of “particular others”, for whom we are responsible [17]. Virginia Held has argued for care ethic’s primacy in health care practice, but also in health care management and policy. She holds, that an ethics of care is suitable for health care policy, as the norms and values that should govern health care providing are the ethical principles of caring for others and doing good for them, not the strive for maximizing profit [17].

Studies have investigated middle-level health care managers’ dealing with ethical conflicts of interest in their work [18, 19]. These studies argued for the need for *ethical competence* in both clinical work and management in health care. Ethical competence has been defined in many ways, but central aspects are the ability to identify ethical values at stake in a particular situation, as well as ethical dilemmas, and to be familiar with different models for weighing interests and values against each other, as well as to have a language that can capture the ethical dimensions of one’s work [20]. Further, it has been argued that an ethical competence must also include the ability to act upon one’s ethical judgements [21]. Also, character traits, i.e., a form of virtue ethics, has been emphasized in the development and practice of ethical competence [21, 22].

## Method

Against this background, the aim of the present study was to investigate perceptions of the status of ethics in the daily work of politicians, chief civil servants and CEOs from care-giver organizations in the county council of Stockholm. An explorative qualitative method was used.

### Participants

Data was collected through individual interviews. In order to get a variation in the data, a strategic selection of informants was made. The purpose was to get a variation concerning age, gender, occupation and experience. All in all, ten representatives from each of the three key groups of influential actors in the county were contacted: county council politicians, chief civil servants and CEOs from care giver organizations. Thirteen of these - five men and eight women - accepted and were included in the study. Drop outs were due to lack of time or lack of interest in the study. Three informants represented county council politicians from different political parties, four were chief civil servants and five were CEOs from different care provider organizations, of which three were publicly and two were privately run.

### Procedure

The informants were recruited by e-mail. Through public web-sites, key actors with much influence in the region, were identified, contacted and asked to participate. Inclusion criteria were that they had influential positions in the county council and had experience of making decisions relevant for the study.

The participants were interviewed by one of the authors (EF) in 2014 and 2015. The interviews were made on places chosen by the informants, in most cases in their work-places. Each interview lasted for about 60–80 min.

A semi-structured interview guide was developed. It covered questions on how ethical aspects were integrated in the informants’ work and specific tasks; how ethically challenging situations were handled; how ethical codes and guidelines were integrated in decision-making processes; the informants’ perceptions of the need for ethical competence in their work and, finally, how they thought they could best develop such competence. The guide was pilot-tested on one person, working as a politician in the county council. The guide worked well and only minor revisions were made.

### Analysis

The interviews were recorded and transcribed verbatim. Data was analyzed through inductive content analysis, as described by Elo & Kyngäs [23]. Initially, the interviews were read through thoroughly in order to find meaning-units covering the research questions. Both authors read transcripts and made the initial coding. Thereafter, they met and compared and discussed their coding and started the categorization, where identified quotations were sorted into categories. The final version of the analysis was made in consensus and agreed upon by both authors.

### Ethical considerations

According to Swedish legislation, no formal approval from the Regional Ethics Review Board was needed for this project, as it did not deal with sensitive, personal data or risked impact the participants physically or psychologically [24]. However, the ethics of scientific work, as outlined in the Declaration of Helsinki [25], was followed in all steps of the work. The informants were included after verbal informed consent. Information about the study was given both verbally and in writing. The participants were informed that participation was voluntary and that they were free to withdraw at any time. The information also emphasized that data would be handled confidentially and that the reporting of results should be done so that identification of the informants was not possible.

## Results

Through the content analysis the following categories were developed: *Low status of ethics; Cost-effectiveness over ethics; Separation of ethics from management*; and *Lack of opportunities for ethical competence building*. In the following, each theme will be illustrated by quotes.

### Low status of ethics

One theme in the interviews concerned the fact that the interviewees apprehended ethics to be of low value in their work. Although the county council of Stockholm had accepted an ethical code for their work, ethics was seldom discussed at the management level, according to the informants.

“I would say that the ethical code is hardly ever discussed in our county. The Swedish Health Care Act is the very foundation of our work, and one of the main pillar of that law is that the patient most in need of care should be prioritized. But we never have that type of discussions, I would say.” (Politician 2)

Also, the Swedish Health Care Act had quite a low status, according to some informants.

“My opinion is that the status of the Health Care Act is far too weak. I mean, it should be superior to all other laws. It is the law that should govern all our work within health care, both here in the county and in the whole of Sweden. But, as a matter of fact, that is not the case.” (Politician 1)

### Cost-effectiveness over ethics

Another recurring theme in the interviews was that ethics was trumped by economy in the daily work of the informants. Within this category, the effects of marketization were often described by the informants.

“Other things govern our work, /---/ mostly, it’s economy that rules today.” (Politician 1)

“There’s a lot of focus on hard facts. /---/ Economism, number of health care visits, and volumes of this and that.” (CEO 1)

Some informants mentioned that ethical values were expressed in the preambles of the county council’s budget, but their impression was that this did not really influence their regular work with the county’s health care budget.

“There are expression of ‘to do’ in the budget, in the preamble, and that could be regarded as an ethical value foundation. Of course, some ethical values can be seen there, as the preamble mentions equal care and all that. But in other respects, these issues are not discussed.” (Politician 2)

Some informants had the opinion that the situation had changed because of the current NPM-influenced organization. They expressed that ethical discussions had been more frequent before the introduction of this system.

“The Health Care Act has a very weak position. It’s not discussed and referred to. We did that ten years ago, in this county. Then we discussed it, but not today.” (Politician 1)

### Separation of ethics from management

Another theme found in the interviews concerned how ethics appeared to be separated from management tasks and referred to either the clinical level or to groups and committees further down in the organization.

“When decisions reach this level, we don’t have ethical discussions. It’s rather a lot of economical debates on how to make ends meet.” (Politician 3)

Likewise, some informants described how ethics was separated from their main tasks, i.e., management work.

“Well, I sit here and take these gigantic decisions, concerning billions of Swedish crones… And then it’s not about the ethics in this decision, or ethical consequences of it, but rather… how much does this cost? /---/ Can we change it and make it … less expensive?” (Politician 1)

Further, the focus on measurements, economic profit and administrative control systems was described as causing ethical dilemmas, since these demands took time and resources that could have been used to meet patients’ needs.

We measure the wrong things! Things that don’t affect the actual care of patients. It’s really useless! (CEO 2)

### Lack of opportunities for ethical competence building

The last category captured expressions of how the informants apprehended that they lacked possibilities for applying ethical competence, as well as competence development when it came to ethics. No forum existed where ethical analyses and discussions could be performed on a regularly basis, in order to improve the informants’ ethical competence, be they CEOs, politicians or civil servants. When ethical competence development was mentioned, the informants rather referred to single, unique opportunities, often related to new regulations or guidelines. But no work-integrated learning opportunities, or comprehensive courses or classes in ethics, were mentioned.

“We have made temporary efforts, such as to give possibilities to go deeper into the question of priority setting a couple of years ago. And the national ethical platform [for priority setting] has been presented on some occasions.” (Politician 2)

Some informants further expressed that regular ethical discussions took place a couple of years ago, but that such opportunities had been reduced in the current system.

“Well, to work with ethics more systematically… we don’t do that anymore, as I see it.” (Chief civil servant 3)

## Discussion

The aim of this study was to explore perceptions of the status of ethics in the daily work of CEOs, politicians and chief civil servants in the county council of Stockholm. The content analysis resulted in four categories: *Low status of ethics; Cost-effectiveness over ethics; Separation of ethics from management*; and *Lack of opportunities for ethical competence building*.

Although ethical values are frequently mentioned and emphasized in Swedish health care policy documents - as described in the Background section - the informants had the impression that in their daily work, ethics had quite a low status. They described how they only sporadically reflected over ethics in their work tasks, be they budgeting, reform work or other decision-making. However, the participants also expressed the importance of ethics in the health care management organization and that they would like to discuss ethical issues to a larger extent; for example, the ethical values in the Health Care Act [1] and in the regional ethical code [12].

Further, the informants described how they often felt a pressure to prioritize administrative and economic goals and requirements linked to market-principles and norms in the management control system, and that this often trumped ethics in their daily work. This is in line with previous research on middle-level health care managers’ experiences of handling conflicts of interest in their work [18, 19, 21], and can be interpreted in light of Max Weber’s concepts of purposive/instrumental rationality and value rationality [26]. As was the case in the study of middle-level health care managers [18, 19], the informants in our study seemed to have difficulties in striking a value balance in their work, resulting in a prioritization of purposive/instrumental rationality over value rationality.

The results concerning how cost-effectiveness was prioritized over ethics can also be analyzed in light of the ethics of care [17]. The informants expressed concerns over their focus on economic results and profit, rather than the ethical principle of caring for other people. Such concerns are in line with Virginia Held’s argument, that the value that should govern health care providing is the ethical principle of care, not the strive for maximizing profit [17].

The informants seemed to fail in integrating ethics in their work processes. This was so, even though the county council had developed an ethical code, intended to guide all health care providing in the county [12]. That ethical codes and guidelines are not always helpful in practical situations in health care work has previously been reported [27, 28].

The analysis also revealed a separation of ethics from the management level, as ethical judgments were not part of the health care managers’ regular work. Thereby, ethics was separated from ordinary work processes, such as budgeting, reform work and formulating agreements between the county (as buyers of health care services) and care providers (such as hospitals and primary care centers). This is in line with Sahlin and Eriksson-Zetterquist [29], who argue that policy and practice, as well as means and ends, are often decoupled in the management of complex organizations. Our results indicate that health care governance is such a complex organization, where ethics and management are separated from each other. According to the informants, this led to the consequence that ethics, although prescribed in policy documents, was not integrated in the actual management of the organization. According to the informants, the separation of ethics from management had increased due to the marketization of the health care system [2, 3]. These findings are in line with previous critique of NPM-like systems in health care organizations [4, 5].

The ethical code for the county council of Stockholm [12] states that the county council should establish possibilities for health care professionals to work according to the ethical code, and that the fundamental values that the code prescribes should be inscribed into agreements and contracts concerning health care providing. According to the informants, this goal was not achieved. Rather, they described how they rarely discussed ethics in their meetings and how decisions taken on their level were not discussed in ethical terms. As mentioned in the background section, the code also states that to use financial resources efficiently is an expression of justice. The reason behind this statement might be that health care resources mainly come from tax payers, and it is assumed that their money should be handled with respect, and not wasted on inefficient treatments for people not in need for them. The respondents in our study seemed to agree with this statement in the code, but expressed concerns over the difficulty to achieve that goal.

Concerning priority-setting, the National Indicators for Good Health Care, [10] produced by the National Board of Health and Welfare, prescribes a division of responsibility; namely, that health care personnel are responsible for fulfilling the principle of human dignity, whereas the providers of care (i.e., hospitals or primary care centers) are responsible for the principle of need and solidarity. The management level, finally, is responsible for the principle of cost-effectiveness. Our results confirm this division, as our informants, representing the management level, described how they emphasized cost-effectiveness over other ethical values, such as solidarity and human dignity. Arguably, cost-effectiveness is an important ethical value. However, the informants in our study seemed to interpret this concept as economic profit and focus on market-principles, rather than as ethical considerations concerning efficient use of scarce resources. Further, it could be argued that the division made in the National Indicators for Good Health Care [10] is problematic, as other regulations, such as those for priority-setting, emphasize that the principles of human dignity, need and solidarity should be considered on *all* levels of health care providing, also the management level. Apparently, the division described in the National Indicators for Good Health Care, [10] created ethical difficulties for the participants in our study, representing the management level.

Finally, our results also showed that ethics was separated from competence development and learning processes, according to the informants. When education or competence development in ethics was mentioned, it was referred to separate, unique occasions, related to new guidelines or regulations. More regular possibilities for work-based ethical competence development were lacking, as well as integrated ethical judgements in the managers’ daily work. The informants further described how such opportunities had occurred a couple of years ago, but that they were lacking in the current system. Previous works have emphasized the need for regular ethical discussions in the development of ethical competence [20, 21]. Eriksson et al. [22] argued that such regular training can contribute to a broad ethical competence, including aspects of virtue ethics as well as utilitarian and deontological ethics.

## Conclusions

Swedish health policy documents include a number of ethical statements and values, but our results showed that in spite of this, ethics had quite a low status in the daily work of the studied representatives of health care management. The interviewed politicians, civil servants and CEOs expressed difficulties in fulfilling the ethical requirements of various policy documents. They described how they prioritized financial requirements above ethics and separated ethics from tasks in their daily work. According to the informants, this development had been enforced by the marketization of the health care system.

Although ethics has a high status in theory, our results indicate that it has a lower status in practice in health care management. In order to change this, and make the requirements in Swedish health care policy documents real, ethical considerations and analyses need to be integrated in the managers’ regular tasks, such as decision-making, budgeting and reform work. Further, opportunities for ethical dialogues on a regular basis should be organized on the management level, in order to increase the managers’ ethical competence. New steering forms, less focused upon market principles, might also be needed, in order to improve the status of ethics in the health care management organization.

## Abbreviations

CEO: Chief Executive Officer
NPM: New Public Management
